# A New Approach to Detecting Atrial Fibrillation Using Count Statistics of Relative Changes between Consecutive RR Intervals

**DOI:** 10.3390/jcm12020687

**Published:** 2023-01-15

**Authors:** Szymon Buś, Konrad Jędrzejewski, Przemysław Guzik

**Affiliations:** 1 Institute of Electronic Systems, Faculty of Electronics and Information Technology, Warsaw University of Technology, Nowowiejska 15/19, 00-665 Warsaw, Poland; 2 Department of Cardiology-Intensive Therapy and Internal Disease, Poznan University of Medical Sciences, 60-355 Poznan, Poland

**Keywords:** atrial fibrillation, cardiac arrhythmia, cardiac time series, electrocardiography, heart rate variability, RR intervals

## Abstract

Background: The ratio of the difference between neighboring RR intervals to the length of the preceding RR interval (x%) represents the relative change in the duration between two cardiac cycles. We investigated the diagnostic properties of the percentage of relative RR interval differences equal to or greater than x% (pRRx%) with x% in a range between 0.25% and 25% for the distinction of atrial fibrillation (AF) from sinus rhythm (SR). Methods: We used 1-min ECG segments with RR intervals with either AF (32,141 segments) or SR (32,769 segments) from the publicly available Physionet Long-Term Atrial Fibrillation Database (LTAFDB). The properties of pRRx% for different x% were analyzed using the statistical procedures and metrics commonly used to characterize diagnostic methods. Results: The distributions of pRRx% for AF and SR differ significantly over the whole studied range of x% from 0.25% to 25%, with particularly outstanding diagnostic properties for the x% range of 1.5% to 6%. However, pRR3.25% outperformed other pRRx%. Firstly, it had one of the highest and closest to perfect areas under the curve (0.971). For pRR3.25%, the optimal threshold for distinction AF from SR was set at 75.32%. Then, the accuracy was 95.44%, sensitivity was 97.16%, specificity was 93.76%, the positive predictive value was 93.85%, the negative predictive value was 97.11%, and the diagnostic odds ratio was 514. The excellent diagnostic properties of pRR3.25% were confirmed in the publicly available MIT–BIH Atrial Fibrillation Database. In a direct comparison, pRR3.25% outperformed the diagnostic properties of pRR31 (the percentage of successive RR intervals differing by at least 31 ms), i.e., so far, the best single parameter differentiating AF from SR. Conclusions: A family of pRRx% parameters has excellent diagnostic properties for AF detection in a range of x% between 1.5% and 6%. However, pRR3.25% outperforms other pRRx% parameters and pRR31 (until now, probably the most robust single heart rate variability parameter for AF diagnosis). The exquisite pRRx% diagnostic properties for AF and its simple computation make it well-suited for AF detection in modern ECG technologies (mobile/wearable devices, biopatches) in long-term monitoring. The diagnostic properties of pRRx% deserve further exploration in other databases with AF.

## 1. Introduction

Many various heart rate variability (HRV) parameters are used alone or in combinations to distinguish atrial fibrillation (AF) from sinus rhythm (SR) [1,2,3]. We and some other authors have shown that the percentage of successive RR interval differences equal to or greater than 50 ms (pRR50) outperforms other HRV parameters for this purpose [4]. Furthermore, machine learning (ML)-aided AF detection using combinations of HRV parameters gives the best results when pRR50 is included in all diagnostic models [2].

The difference between the durations of consecutive cardiac beats (x) at least 50 ms is one of several possible x values. After studying a wide range of such x differences between 7 ms to 195 ms, we recently demonstrated that other pRRx parameters had better diagnostic properties for AF detection than pRR50. In over 60,000 electrocardiograms (ECGs) of 1-min duration with either SR or AF, pRR31, i.e., pRRx for x = 31 ms, was the most effective for AF diagnosis. We reported that the diagnostic odds ratio (DOR) in AF detection was 194 for pRR31, which significantly outperformed pRR50 (DOR of 173) [3].

**Figure 1:**
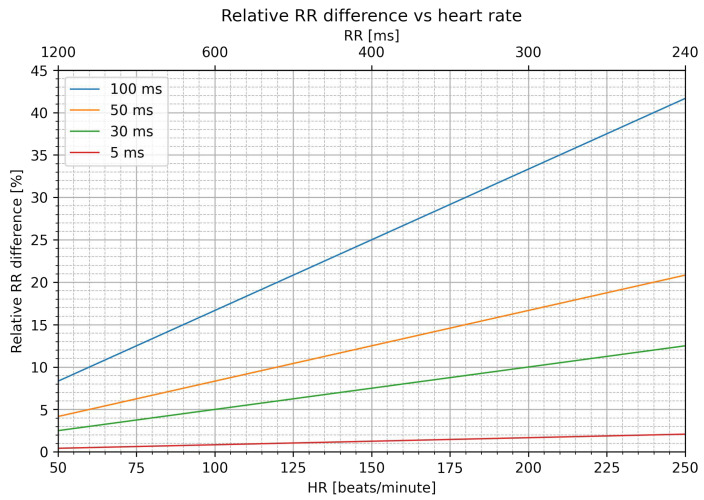
Relationship between heart rate (HR) and relative difference between consecutive RR intervals for absolute differences equal to 5, 30, 50, and 100 ms.

pRRx quantifies the percentage of consecutive RR intervals differing by at least x ms, where x is the absolute difference between two consecutive RR intervals. As Figure 1 shows, the relative effect of x, such as 50 ms, varies depending on the duration of the preceding RR interval. For example, a difference of 50 ms between two RR intervals that are close to 500 ms (heart rate–HR–of 120 beats/min) corresponds to approximately 10% of the relative difference, whereas the same difference between two RR intervals that are closer to 1200 ms (HR of 50 beats/min) corresponds to only 4.2% of the relative difference. The relative difference between RR intervals increases linearly with heart rate.

Instead of measuring the absolute difference between two consecutive RR intervals with x, it is possible to use a relative measure of such difference, the ratio of the difference to the duration of the preceding RR interval (x%). The percentage of relative RR interval differences equal to or greater than x% (pRRx%) describes this measure. pRRx% indicates how many RR intervals differed from the previous RR interval by at least x% of its length.

In the past, RRx% (from which pRRx% is derived) has been occasionally studied. Ewing et al. [5] proposed the total count of successive RR intervals that differ by at least x = 50 ms (RR50) and by at least x = 6.25% of the previous RR interval (RR6.25%). They used these parameters to distinguish between various conditions or groups of subjects. For example, they found differences between those who were sleeping versus awake, or subjects who were healthy versus diabetic or heart transplant patients. RR50 (and pRR50) [6]), has been widely adopted in HRV analysis [1]. However, neither RR6.25% nor the whole RRx% and pRRx% families have received much attention in the HRV field. The diagnostic properties of pRRx% for AF detection have not been studied in detail before.

In this study, we aimed to investigate the diagnostic properties of pRRx% for AF detection in a wide range of x% in 1-min ECGs with either SR or AF. Additionally, we directly compared pRR31 and the best-found pRRx% to better discriminate between SR and AF.

## 2. Materials and Methods

### 2.1. Data

The threshold values x of pRRx% parameters investigated in the paper ranged from 0.25% to 25% of the previous RR interval. The step of changes was 0.25% which gives 100 parameters in total. We used the same dataset of RR intervals as in our previous publication. We used data from the Long-Term Atrial Fibrillation Database (LTAFDB) [7,8], which contains 84 Holter electrocardiographic (ECG) recordings with an average duration of 24 h, sampled at 128 Hz. The database includes information about R-wave locations, the type of corresponding cardiac beats, and the onset of different cardiac rhythms. We used the database’s R-wave location information and rhythm onset information and included only uninterrupted 1-min ECG segments with AF or SR, excluding segments with other rhythms. We applied the same preprocessing approach as in our previous study, dividing the original and continuous RR interval series into 1-min, non-overlapping segments containing only SR or AF [3].

To minimize unidentified technical artifacts, we removed RR intervals shorter than 240 ms (corresponding to HR > 250 beats/min) or longer than 3000 ms (corresponding to HR < 20 beats/min). Altogether, 0.45% of all RR intervals were removed. We have also used another rejection criterion for each of the 1-min segments. If the total length of the removed RR intervals in a 1-min segment exceeded 6 s, we excluded the whole segment from the analysis.

### 2.2. Software Tools

We used Python programming language (version 3.9, Python Software Foundation, Wilmington, DE, USA) for all the analyses. For receiver operating characteristic analysis, we used scikit-learn library (version 1.3.3) [9]. For statistical analysis, we used Numpy library (version 1.20.3) [10]. The source code for all the analyses in the study is available at https://github.com/simonbus/prrx_af (accessed on 11 January 2023).

### 2.3. Statistical Analysis

We used the d’Agostino–Pearson test to formally analyze the data distribution of pRRx% for each x% in the range of 0.25% to 25%. Neither the SR nor the AF data had normal distributions of pRRx%. We plotted selected percentiles (10th, 25th, 50th, 75th, and 90th) of the pRRx% distributions in AF and SR as functions of the threshold x%. We then used receiver operating characteristic (ROC) analysis to compute the area under the curve (AUC) for AF detection for all studied pRRx% and identified the optimal cutoff value using Youden’s index to maximize the sum of sensitivity and specificity for AF/SR discrimination [11,12]. For each pRRx%, we calculated classification metrics such as accuracy, sensitivity, specificity, positive predictive value (PPV), negative predictive value (NPV), and diagnostic odds ratio (DOR) for distinguishing AF from SR using the optimal cutoff [13]. To estimate the 95% confidence interval (CI) for each classification metric, we used nonparametric bootstrap with 5000 samples [14]. Additionally, we compared the diagnostic properties of the pRR31 parameter (the best among pRRx [3]) against the best pRRx% from the perspective of AF detection.

## 3. Results

### 3.1. Data Distribution Analysis

**Figure 2:**
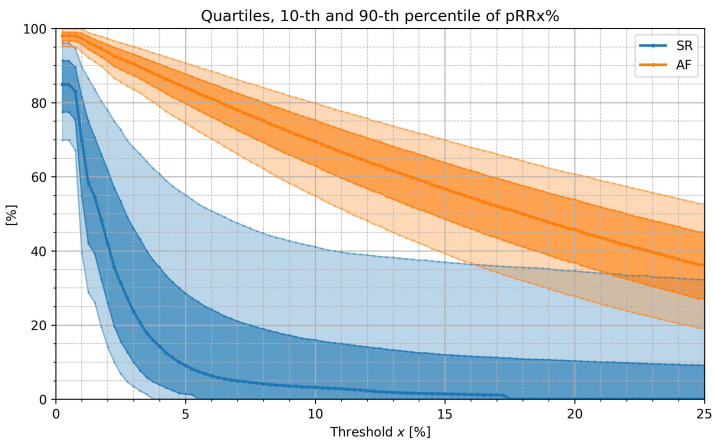
Medians (bolder line), interquartile ranges (darker band), and 10th to 90th percentile ranges (lighter band) of the percentages of successive RR intervals differing by at least x% of the previous RR interval (pRRx%) for sinus rhythm (SR) and atrial fibrillation (AF) for different x thresholds.

Figure 2 presents medians (bolder line), the interquartile range (darker band), and the 10th to 90th percentile range (lighter band) of pRRx% distribution in SR (blue) and AF (orange). Median values of SR and AF never cross or overlap in the whole range of studied x. The same is true for the 75th percentiles of pRRx% in SR and the 25th percentiles of pRRx% in AF (no overlap between the darker bands). Additionally, the 90th percentile of pRRx% in SR does not cross with the 10th percentile in AF in the range of x from 0.75% to 16% (white space between the lighter bands). It indicates that there is a broad range of x thresholds of pRRx% with very good diagnostic properties in AF/SR distinction and suggests that the optimal value of x lies within this range.

### 3.2. Area under ROC Curve (AUC)

**Figure 3:**
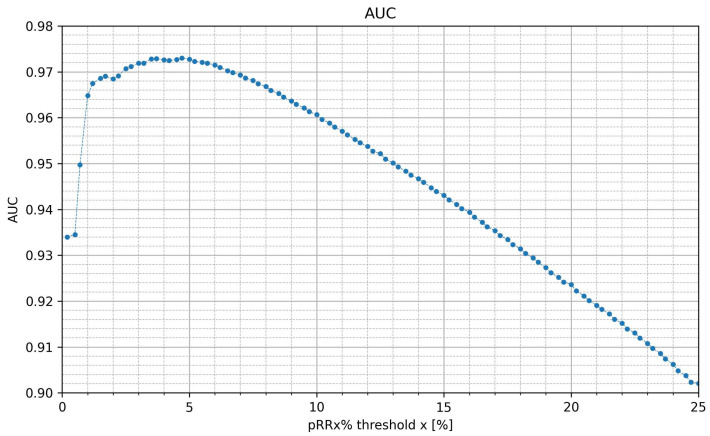
Area under curve (AUC) of the percentages of successive RR intervals differing by at least x% of the previous RR interval (pRRx%) for the distinction of AF from SR as a function of the x threshold.

The values of AUC for the AF/SR distinction, shown in Figure 3, exceed 0.96 for x in the range of 1% to 10%, and exceed 0.97 for x in the range of 2.5% to 7.5%. AUCs gradually decrease to 0.90 for the highest x thresholds. In other words, pRRx% for a wide range of moderate x values has excellent AUC (exceeding 0.9) for distinguishing the SR from AF recordings. The maximal AUC value, considered as one of the criteria for selecting the optimal threshold, was 0.973 for x = 4.75%, i.e., pRR4.75% for differentiating SR from AF with pRRx%. However, very similar values of AUC occur in the whole range of 2.5% to 7.5%.

### 3.3. Optimal Cutoff Values

**Figure 4:**
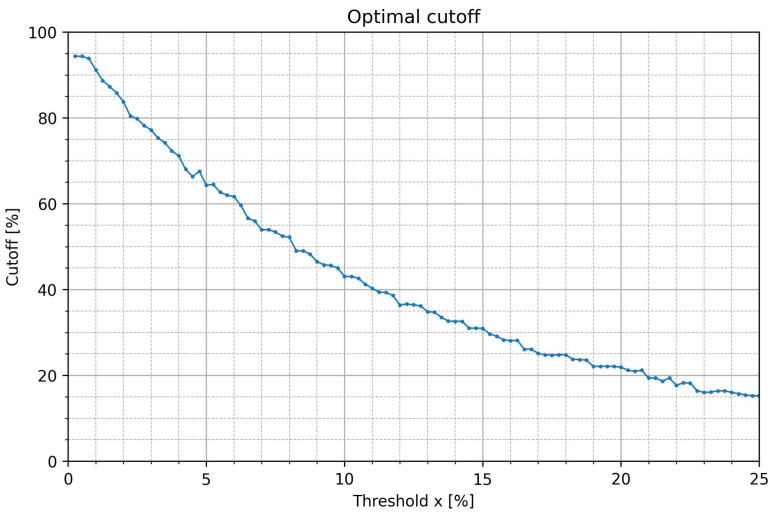
Optimal cutoff values based on the Youden index for the percentages of successive RR intervals differing by at least x% of the previous RR interval (pRRx%) for the distinction of AF from SR as a function of the x threshold.

Table 1 summarizes optimal cutoffs for all studied pRRx% (Figure 4). The cutoffs generally decrease as the threshold x increases, from 94.35% (pRR0.25%) to 15.27% (pRR25%).

### 3.4. Classification Metrics

**Figure 5:**
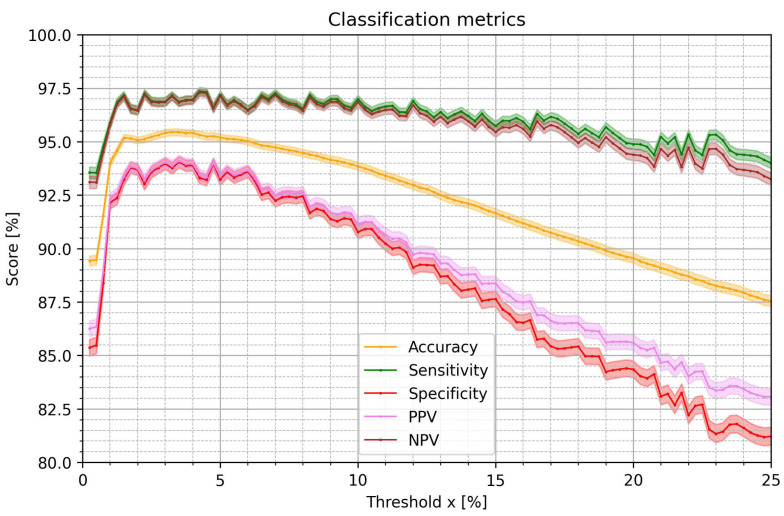
Medians and 95% confidence intervals of diagnostic classification metrics for optimum cutoff values for the percentages of successive RR intervals differing by at least x% of the previous RR interval (pRRx%) for the distinction of AF from SR as a function of the x threshold. PPV—positive predictive value, NPV—negative predictive value.

The metrics of AF detection with a single pRRx% parameter and the optimal cutoff value were estimated with the nonparametric bootstrap. Figure 5 presents medians and 95% CI ranges for accuracy, sensitivity, specificity, positive predictive value (PPV), and negative predictive value (NPV). All the metrics increase rapidly for x between 0.25% and 1.25%, achieve the highest values for x in the range between 1.25% and 7%, and then gradually decline. Accuracy is highest for pRR3.25% (median 95.44%). Sensitivity is highest for pRR4.25% (median 97.36%). Specificity is highest for pRR3.5% (median 94.05%). PPV is highest for pRR3.5% (median 94.10%). NPV is highest for pRR4.25% (median 97.30%). For each classification metric, we used a *t*-test to compare the distributions for different parameters. All the differences were statistically significant, except for the difference between PPV for pRR3.25% and pRR2.75% (*p*-value = 0.947).

### 3.5. Diagnostic Odds Ratios

**Figure 6:**
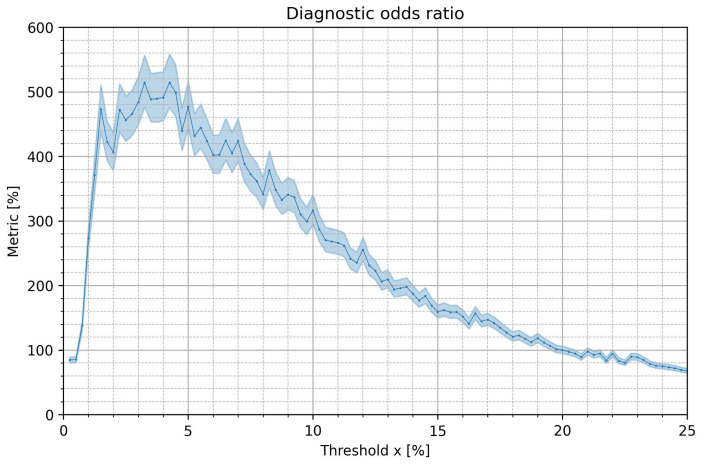
Medians and 95% confidence intervals of diagnostic odds ratio for optimum cutoff values for the percentages of successive RR intervals differing by at least x% of the previous RR interval (pRRx%) for the distinction of AF from SR as a function of the x threshold.

In Figure 6, median values and 95% of DOR are presented for optimal cutoff values of pRRx%. DOR is 84.67 for pRR0.25%, increases to 514.48 for pRR4.25% and then gradually decreases to 67.73 for pRR25%. DOR exceeds 400 for x in the range of 1.5% to 7%. We compared distributions of DOR for different pRRx% using a *t*-test. All the differences were statistically significant, except for the differences between pRR2.25% and pRR1.5% (*p*-value = 0.755) and between pRR3.25% and pRR4.25% (*p*-value = 0.966). In the latter case, it means that both pRR4.25% and pRR3.25% may be equally considered as having the highest DOR, i.e., 514.48 and 514.40, respectively.

### 3.6. Comparison of pRR31 and pRR3.25%

**Figure 7:**
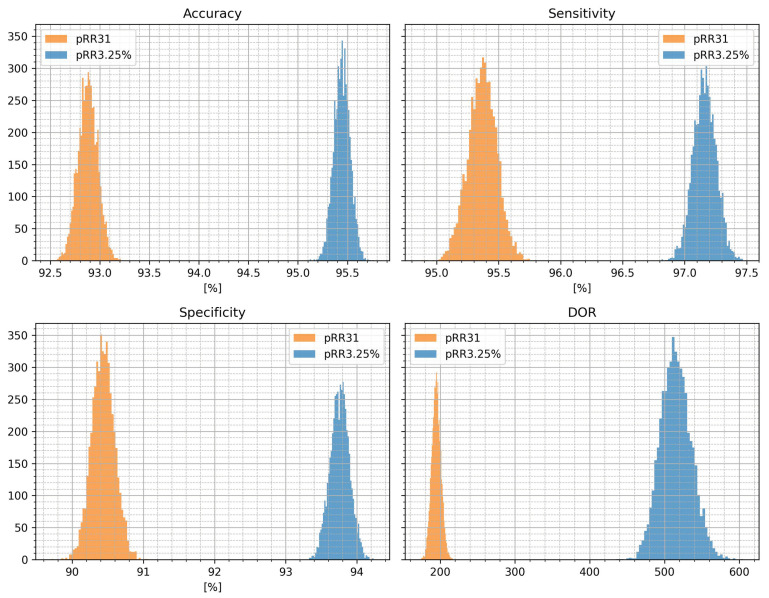
Histograms of classification metrics for optimum cutoff values for pRR31 and pRR3.25% (the percentages of successive RR intervals differing by at least 31 ms and 3.25% of the previous RR interval, respectively). DOR—diagnostic odds ratio.

The nonparametric bootstrap was used to compare the distributions of classification metrics for pRR31 and pRR3.25%. Figure 7 shows histograms of accuracy, sensitivity, specificity, and DOR for comparing pRR31 and pRR3.25%. Although using x = 31 ms (pRR31) helps improve all metrics compared to x = 50 ms (pRR50) [3], the x = 3.25% (pRR3.25%) even further improves the distinction between 1 and min ECGs with AF and SR. The histograms demonstrate that the better performance of pRR3.25% is not random because there is no overlap between distributions of classification metrics for pRR31 and pRR3.25%.

## 4. Discussion

This study demonstrated that the pRRx% family could effectively discriminate AF from SR in 1-min ECGs across a wide range of x%. The pRRx% in the range of 1.5% to 6% showed exceptional diagnostic properties, with pRR3.25% outperforming other pRRx% in this range. pRR3.25% had better diagnostic properties than pRRx for x = 31 ms, which has previously been the best-performing HRV index for distinguishing AF from SR. The median accuracy of pRRx% for x% in the range of 1.5% to 6% exceeded 95%. pRR3.25% had one of the highest areas under the curve (AUC) at 0.971 and the highest accuracy. An AUC close to 1 indicates that a particular parameter is nearly perfect for diagnostic or predictive purposes. Therefore, pRR3.25% appears to have excellent properties for AF diagnosis in 1-min ECGs. If the number of successive RR intervals differing by more than 3.25% exceeds 75.32% (i.e., three-quarters of all RR intervals) in a 1-min ECG, it is most likely to be AF rather than SR. The median accuracy for the 75.32% threshold is 95.44%, with sensitivity at 97.16%, specificity at 93.76%, positive predictive value (PPV) at 93.85%, negative predictive value (NPV) at 97.11%, and a diagnostic odds ratio of about 514. No other HRV indices studied to date have shown similar diagnostic properties for AF detection.

As shown in Figure 7, pRR3.25% (relative threshold 3.25%) outperforms pRR31 (absolute threshold 31 ms), which has previously been the best single HRV parameter for differentiating AF from SR [3]. In the same dataset, the Long-Term Atrial Fibrillation Database (LTAFDB), pRR31 had accuracy, sensitivity, and specificity values exceeding 90% (92.86%, 95.37%, and 90.42%, respectively), which is 2–3.5% worse for each respective metric than for pRR3.25%. This improvement is clinically meaningful, especially since all metrics are above 90%. Additionally, there is a significant difference in the diagnostic odds ratios between pRR3.25% and pRR31, which are 514 and 194, respectively. Noteworthily, pRR31 has much better classification measures for distinguishing AF from SR than pRR50, which is the most common pRRx parameter for AF detection.

### 4.1. Potential Applications

HRV indices have been used for automatic AF detection and screening in ECGs or photoplethysmography signals of arterial pressure waveforms. They have become helpful in various medical and non-medical technologies, e.g., wearable devices recording ECG continuously or on demand. Newer Holter ECG recorders may record ECG for up to 14 days, whereas the in-hospital monitoring systems work for the whole patient’s stay. Various implantable devices, such as implantable loop recorders, pacemakers, implantable cardioverter-defibrillators (ICDs), and cardiac resynchronization therapy devices (CRTDs) continuously monitor intracardiac electrograms for arrhythmias. They store suspicious ECG segments based on real-time analysis with built-in algorithms. Most photoplethysmography-based devices for HR monitoring use HRV algorithms to detect AF. Several international consensus documents and guidelines have accepted the newer technologies, based on HRV analysis, for the screening and detection of AF [15,16,17,18,19,20,21,22,23,24,25].

Mobile and wearable technologies using single-lead ECG (from touch ECG recorders or selected smartwatches) or photoplethysmographic signals have shown sensitivities of 91.5–99% and specificities of 76–100% for AF detection, compared to standard 12-lead ECG [16]. HRV indices are employed for fast preselection of ECG segments with AF in very long ECG recordings lasting up to several weeks.

Newer wearable technologies have several advantages over standard 12-lead ECG. They may be used in special conditions, such as during physical work, training, or competitive sports. The ECG recording might last up to several weeks. However, these technologies also have some disadvantages. Less evidence supporting their clinical utility exists. Such recordings have more, usually motion-related, technical artifacts. A massive amount of data collected by long-term recorders has become an analytical challenge [19].

The 12-lead ECGs are typically 8 to 12 s long. The 12-lead ECG is considered the gold standard for diagnosing AF, but only if it is acquired during an AF episode. The 24-h ECG Holter monitoring is the preferred method if AF is paroxysmal. Unfortunately, AF occurs infrequently in many patients. Longer ECG monitoring, up to 4 weeks, may be necessary, like in patients with cryptogenic stroke.

AF identification from a standard short 12-lead ECG is usually instant for clinicians. Visual inspection and analysis of 24–48 h of Holter ECG data typically take 10–30 min. It is practically impossible to use a similar approach for recordings lasting several days, weeks, or even months. On average, a 24-h ECG contains between 80,000 and 120,000 RR intervals, whereas longer recordings contain millions of RR intervals, falling into the realm of Big Data. Thus, the automatic screening of suspicious ECG segments and their preselection for human visual inspection is a fundamental necessity. We are in the early stages of using Big Data analysis in clinical medicine, and there is a need for fast and easily applicable computational algorithms to analyze such data.

The computation of pRRx% is straightforward, making it particularly well-suited for the following applications:

- mobile and wearable devices, such as ECG patches, vests, and Holter ECG recorders with built-in algorithms;
- monitoring systems analyzing online ECGs in hospitals or remotely via telemetry;
- algorithms in smartphones and smartwatches analyzing short-event ECGs on demand;
- portable ECG machines for medical staff, recording ECGs at the point of care;
- implantable devices continuously monitoring ECGs and searching for specific events, such as implantable loop recorders, pacemakers, ICDs, and CRTDs.

Using pRRx% might increase the AF detection performance in ECG recordings of any length, from minutes to weeks. It might also simplify the complexity of software or hardware solutions. We acknowledge that the utility of pRRx% for AF detection should be prospectively investigated on a large scale, preferably in multicenter studies.

### 4.2. Study Limitations

**Figure A1:**
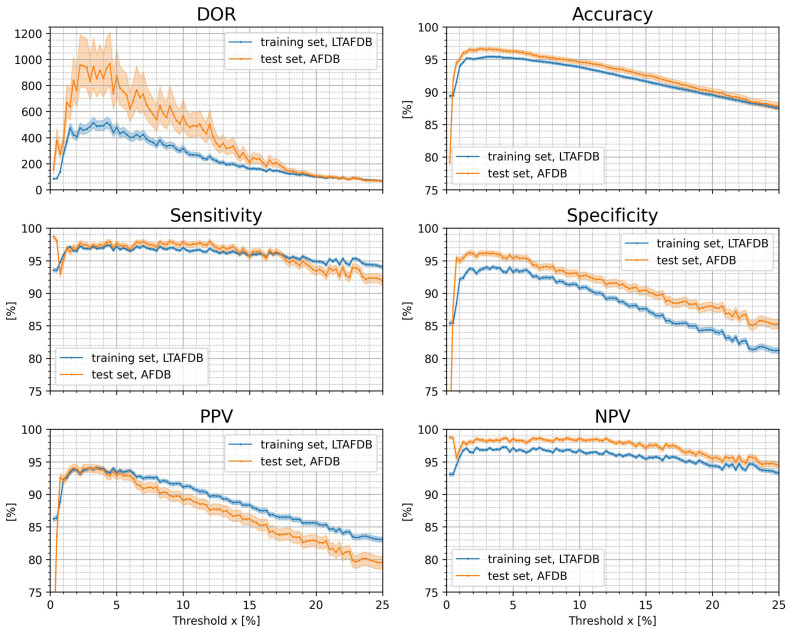
Medians and 95% confidence intervals of AF detection results in the training set Long-Term Atrial Fibrillation Database (LTAFDB) and the test set MIT-BIH Atrial Fibrillation Database (AFDB): diagnostic odds ratio (DOR, **top left**), accuracy (**top right**), sensitivity (**middle left**), specificity (**middle right**), positive predictive value (PPV, **bottom left**), and negative predictive value (NPV, **bottom right**).

**Figure A2:**
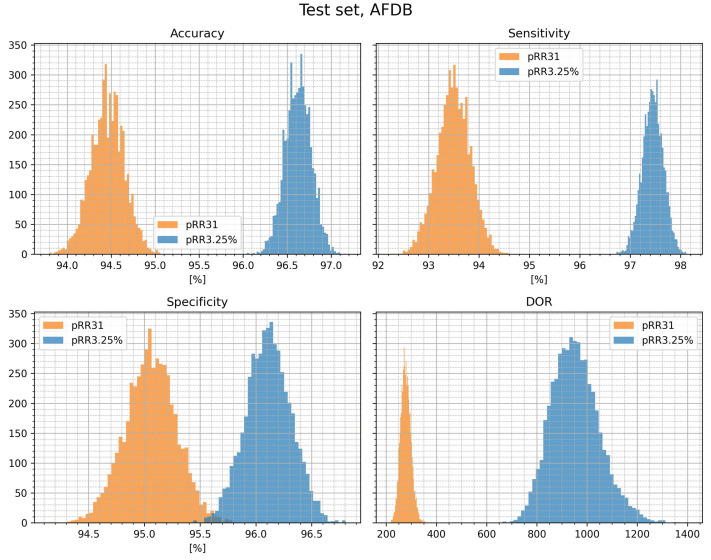
Histograms of atrial fibrillation detection results in the test set MIT-BIH Atrial Fibrillation Database (AFDB) for pRR31 and pRR3.25% (the percentages of successive RR intervals differing by at least 31 ms and 3.25% of the previous RR interval, respectively). DOR—diagnostic odds ratio.

Our study has several limitations. It is an observational and retrospective analysis using ECG recordings from a single database (LTAFDB) [3]. For verification, whether the results are not specific to a single database, we tested the diagnostic value of pRRx% parameters (with cutoffs determined in LTAFDB) using different data from the MIT-BIH Atrial Fibrillation Database (AFDB) [7,26]. The results are presented in Appendix A. In most cases, the classification results in the test set are better than in the training set (Figure A1). The highest DOR was achieved by pRR4.5% (median 967.51), accuracy by pRR2.5% (median 96.64%), sensitivity by pRR0.25% (median 98.68%), and specificity by pRR1.75% (median 96.21%). Importantly, the classification results in the test set using pRR3.25% (as in the training set) are better than those using pRR31 (median accuracy 96.55% vs. 94.45%, sensitivity 97.45% vs. 93.50%, specificity 96.11% vs. 95.05%, DOR 947.82 vs. 276.88) (Figure A2).

We have previously used the same data, consisting of RR intervals from over 60,000 1-min ECG segments with either AF or SR, in our studies [2,3]. In [2], we selected various HRV indices for the machine learning algorithms, whereas in [3], we determined the optimal x for pRRx. Although using the same database for different studies has some limitations, it also has practical benefits. The comparison and cross-validation of various methodological approaches and parameters are more straightforward with the same data.

Another limitation of this study is the arbitrary choice of 1-min ECGs. Recently, we also investigated the diagnostic properties of various HRV parameters based on RR interval analysis for ECGs of lengths between 5 and 300 s and found that some HRV parameters had improved diagnostic properties for AF detection in longer ECG recordings [27]. However, 1-min ECGs are commonly used in other studies and were used in our previous investigations. Switching to ECGs of other lengths could be confusing and complicate comparisons, such as between pRR3.25% and pRR31.

## 5. Conclusions

We have demonstrated that the pRRx% parameters are excellent for differentiating AF from SR using RR intervals from 1 to min ECGs. Additionally, we found that pRRx% is superior to pRRx, at least for 1-min ECGs. Older studies reported that pRR50 is better than other HRV parameters for distinguishing AF from SR. Recently, we found that pRR31 is better than pRR50 for this purpose.

However, with this study, we demonstrate that the pRRx% family even further improves the diagnosis of AF. Of all pRRx%, pRR3.25% currently appears to be the best single HRV-based parameter for differentiating AF from SR. Although the pRRx% parameters show promising results in differentiating AF from SR, further research is needed to confirm their effectiveness in various populations, settings, and medical and non-medical devices.

## Figures and Tables

**Table 1:** Optimal cutoff values based on Youden index for the percentages of successive RR intervals differing by at least x% of the previous RR interval (pRRx%) for the distinction of AF from SR.

Parameter	Cutoff	Parameter	Cutoff	Parameter	Cutoff	Parameter	Cutoff
pRR0.25%	94.35%	pRR6.5%	56.64%	pRR12.75%	36.18%	pRR19.0%	22.07%
pRR0.5%	94.35%	pRR6.75%	55.97%	pRR13.0%	34.84%	pRR19.25%	22.12%
pRR0.75%	93.86%	pRR7.0%	53.95%	pRR13.25%	34.71%	pRR19.5%	22.12%
pRR1.0%	91.22%	pRR7.25%	53.95%	pRR13.5%	33.54%	pRR19.75%	22.07%
pRR1.25%	88.71%	pRR7.5%	53.42%	pRR13.75%	32.63%	pRR20.0%	21.90%
pRR1.5%	87.32%	pRR7.75%	52.45%	pRR14.0%	32.61%	pRR20.25%	21.19%
pRR1.75%	85.84%	pRR8.0%	52.20%	pRR14.25%	32.61%	pRR20.5%	20.95%
pRR2.0%	83.76%	pRR8.25%	49.03%	pRR14.5%	31.00%	pRR20.75%	21.19%
pRR2.25%	80.45%	pRR8.5%	49.01%	pRR14.75%	31.00%	pRR21.0%	19.38%
pRR2.5%	79.81%	pRR8.75%	48.28%	pRR15.0%	30.94%	pRR21.25%	19.42%
pRR2.75%	78.20%	pRR9.0%	46.53%	pRR15.25%	29.66%	pRR21.5%	18.62%
pRR3.0%	77.19%	pRR9.25%	45.75%	pRR15.5%	29.14%	pRR21.75%	19.38%
pRR3.25%	75.32%	pRR9.5%	45.60%	pRR15.75%	28.28%	pRR22.0%	17.69%
pRR3.5%	74.19%	pRR9.75%	45.00%	pRR16.0%	28.10%	pRR22.25%	18.25%
pRR3.75%	72.31%	pRR10.0%	43.05%	pRR16.25%	28.14%	pRR22.5%	18.24%
pRR4.0%	71.17%	pRR10.25%	43.06%	pRR16.5%	26.11%	pRR22.75%	16.41%
pRR4.25%	68.03%	pRR10.5%	42.61%	pRR16.75%	26.11%	pRR23.0%	16.03%
pRR4.5%	66.34%	pRR10.75%	41.25%	pRR17.0%	25.15%	pRR23.25%	16.08%
pRR4.75%	67.55%	pRR11.0%	40.28%	pRR17.25%	24.78%	pRR23.5%	16.41%
pRR5.0%	64.33%	pRR11.25%	39.39%	pRR17.5%	24.73%	pRR23.75%	16.38%
pRR5.25%	64.47%	pRR11.5%	39.34%	pRR17.75%	24.81%	pRR24.0%	16.03%
pRR5.5%	62.66%	pRR11.75%	38.60%	pRR18.0%	24.81%	pRR24.25%	15.71%
pRR5.75%	62.02%	pRR12.0%	36.42%	pRR18.25%	23.76%	pRR24.5%	15.44%
pRR6.0%	61.64%	pRR12.25%	36.62%	pRR18.5%	23.66%	pRR24.75%	15.27%
pRR6.25%	59.63%	pRR12.5%	36.42%	pRR18.75%	23.57%	pRR25.0%	15.27%

## Data Availability

MIT-BIH Atrial Fibrillation Database (AFDB) [7,26], and Long-Term AF Database (LTAFDB) [7,8] were used in the study. They are available at https://physionet.org/content/afdb/1.0.0/ (accessed on 1 December 2022) and https://physionet.org/content/ltafdb/1.0.0/ (accessed on 1 December 2022), respectively.

## Appendix A. Results of Validation on MIT-BIH AF Database

We used the optimal cutoff values of pRRx% parameters from the training set Long-Term Atrial Fibrillation Database (LTAFDB) to detect AF in the test set from the Massachusetts Institute of Technology–Beth Israel Hospital (MIT-BIH) Atrial Fibrillation Database (AFDB) [7,26]. Data preparation was the same as in the training set, and nonparametric bootstrap with 5000 samples was used to estimate the distributions of classification results.

### Appendix A.1. Comparison between Training Set and Test Set Results

Results comparing AF detection in the training set and the test set are presented in Figure A1. The classification metrics include diagnostic odds ratio (DOR), accuracy, sensitivity, specificity, positive predictive value (PPV), and negative predictive value (NPV). In the test set, the highest DOR (median 276) and accuracy (median 0.944) are achieved for pRR31 (in training set 194 and 0.928, respectively). Peak values of DOR, accuracy, PPV, NPV, and specificity are higher in the test set AFDB, whereas sensitivity is higher in the training set LTAFDB in the whole range of threshold values x.

### Appendix A.2. Comparison between pRR31 and pRR3.25%

The histograms in Figure A2 show the distribution of four classification metrics (accuracy, sensitivity, specificity, and DOR) in AF detection in the test set using pRR31 and pRR3.25%. For accuracy, specificity, and DOR, the results are higher for pRR3.25% than for pRR31. For specificity, there is an overlap between the distributions for pRR31 and pRR3.25%, but the average is higher for pRR3.25%.

### Appendix A.3. Conclusions from the Validation

The results from the Appendix confirm the findings of the study. For AF detection in 1-min ECG, using relative thresholds for counting RR interval differences (pRRx%) rather than absolute thresholds (pRRx) is more effective. Both in the LTAFDB training and in the test AFDB sets, the best performance measured by accuracy and DOR are achieved by pRRx% with thresholds in the range 2–5%. pRR3.25% outperforms pRR31, previously shown as the most effective among pRRx parameters [3]. The findings from the training LTAFDB set translate well to the test AFDB set, demonstrating that the effectiveness of pRRx% parameters in AF detection is not limited to a single ECG database.

## References

[1] Electrophysiology Task Force of the European Society of Cardiology the North American Society of Pacing (1996). Heart Rate Variability. Standards of measurement, physiological interpretation, and clinical use. Circulation.

[2] Buś S., Jędrzejewski K., Guzik P. (2022). Using Minimum Redundancy Maximum Relevance Algorithm to Select Minimal Sets of Heart Rate Variability Parameters for Atrial Fibrillation Detection. J. Clin. Med..

[3] Buś S., Jędrzejewski K., Guzik P. (2022). Statistical and Diagnostic Properties of pRRx Parameters in Atrial Fibrillation Detection. J. Clin. Med..

[4] Buś S., Jędrzejewski K., Krauze T., Guzik P. (2020). Feasibility Study on the Use of Heart Rate Variability Parameters for Detection of Atrial Fibrillation with Machine Learning Techniques. Proceedings of the 2020 Signal Processing Workshop (SPW).

[5] Ewing D.J., Neilson J.M., Travis P. (1984). New method for assessing cardiac parasympathetic activity using 24 hour electrocardiograms. Heart.

[6] Bigger Jr J.T., Kleiger R.E., Fleiss J.L., Rolnitzky L.M., Steinman R.C., Miller J.P. (1988). Components of heart rate variability measured during healing of acute myocardial infarction. Am. J. Cardiol..

[7] Goldberger A.L., Amaral L.A., Glass L., Hausdorff J.M., Ivanov P.C., Mark R.G., Mietus J.E., Moody G.B., Peng C.K., Stanley H.E. (2000). PhysioBank, PhysioToolkit, and PhysioNet: Components of a new research resource for complex physiologic signals. Circulation.

[8] Petrutiu S., Sahakian A.V., Swiryn S. (2007). Abrupt changes in fibrillatory wave characteristics at the termination of paroxysmal atrial fibrillation in humans. EP Europace.

[9] Pedregosa F., Varoquaux G., Gramfort A., Michel V., Thirion B., Grisel O., Blondel M., Prettenhofer P., Weiss R., Dubourg V. (2011). Scikit-learn: Machine Learning in Python. J. Mach. Learn. Res..

[10] Harris C.R., Millman K.J., van der Walt S.J., Gommers R., Virtanen P., Cournapeau D., Wieser E., Taylor J., Berg S., Smith N.J. (2020). Array programming with NumPy. Nature.

[11] Fawcett T. (2006). An introduction to ROC analysis. Pattern Recognit. Lett..

[12] Youden W.J. (1950). Index for rating diagnostic tests. Cancer.

[13] Glas A.S., Lijmer J.G., Prins M.H., Bonsel G.J., Bossuyt P.M.M. (2003). The diagnostic odds ratio: A single indicator of test performance. J. Clin. Epidemiol..

[14] Efron B. (1979). Bootstrap Methods: Another Look at the Jackknife. Ann. Stat..

[15] Steinberg J.S., Varma N., Cygankiewicz I., Aziz P., Balsam P., Baranchuk A., Cantillon D.J., Dilaveris P., Dubner S.J., El-Sherif N. (2017). 2017 ISHNE-HRS expert consensus statement on ambulatory ECG and external cardiac monitoring/telemetry. Ann. Noninvasive Electrocardiol..

[16] Hindricks G., Potpara T., Dagres N., Arbelo E., Bax J.J., Blomström-Lundqvist C., Boriani G., Castella M., Dan G.A., Dilaveris P.E. (2021). 2020 ESC Guidelines for the diagnosis and management of atrial fibrillation developed in collaboration with the European Association for Cardio-Thoracic Surgery (EACTS) The Task Force for the diagnosis and management of atrial fibrillation of the European Society of Cardiology (ESC) Developed with the special contribution of the European Heart Rhythm Association (EHRA) of the ESC. Eur. Heart J..

[17] Varma N., Cygankiewicz I., Turakhia M., Heidbuchel H., Hu Y., Chen L.Y., Couderc J.P., Cronin E.M., Estep J.D., Grieten L. (2021). 2021 ISHNE/HRS/EHRA/APHRS Collaborative Statement on mHealth in Arrhythmia Management: Digital Medical Tools for Heart Rhythm Professionals: From the International Society for Holter and Noninvasive Electrocardiology/Heart Rhythm Society/European Heart Rhythm Association/Asia Pacific Heart Rhythm Society. Cardiovasc. Digit. Health J..

[18] Zeppenfeld K., Tfelt-Hansen J., de Riva M., Winkel B.G., Behr E.R., Blom N.A., Charron P., Corrado D., Dagres N., de Chillou C. (2022). 2022 ESC Guidelines for the management of patients with ventricular arrhythmias and the prevention of sudden cardiac death: Developed by the task force for the management of patients with ventricular arrhythmias and the prevention of sudden cardiac death of the European Society of Cardiology (ESC) Endorsed by the Association for European Paediatric and Congenital Cardiology (AEPC). Eur. Heart J..

[19] Duncker D., Ding W.Y., Etheridge S., Noseworthy P.A., Veltmann C., Yao X., Bunch T.J., Gupta D. (2021). Smart Wearables for Cardiac Monitoring—Real-World Use beyond Atrial Fibrillation. Sensors.

[20] Sanchis-Gomar F., Lavie C.J., Perez M.V. (2021). Consumer wearable technologies to identify and monitor exercise-related arrhythmias in athletes. Curr. Opin. Cardiol..

[21] Guzik P., Malik M. (2016). ECG by mobile technologies. J. Electrocardiol..

[22] Gajda R., Biernacka E.K., Drygas W. (2018). Are heart rate monitors valuable tools for diagnosing arrhythmias in endurance athletes?. Scand. J. Med. Sci. Sports.

[23] Gajda R. (2020). Is Continuous ECG Recording on Heart Rate Monitors the Most Expected Function by Endurance Athletes, Coaches, and Doctors?. Diagnostics.

[24] Kalarus Z., Mairesse G.H., Sokal A., Boriani G., Średniawa B., Casado Arroyo R., Wachter R., Frommeyer G., Traykov V., Dagres N. (2022). Searching for atrial fibrillation: Looking harder, looking longer, and in increasingly sophisticated ways. An EHRA position paper’. EP Eur..

[25] Matusik P.S., Matusik P.T., Stein P.K. (2022). Heart rate variability and heart rate patterns measured from wearable and implanted devices in screening for atrial fibrillation: Potential clinical and population-wide applications. Eur. Heart J..

[26] Moody G. (1983). A new method for detecting atrial fibrillation using RR intervals. Comput. Cardiol..

[27] Buś S., Jędrzejewski K., Guzik P. Impact of Electrocardiogram Length on Diagnostic Properties of Heart Rate Variability Indices in Atrial Fibrillation Detection. Proceedings of the 2022 12th Conference of the European Study Group on Cardiovascular Oscillations (ESGCO).

